# Mitral cell development in the olfactory bulb of sharks: evidences of a conserved pattern of glutamatergic neurogenesis

**DOI:** 10.1007/s00429-019-01906-9

**Published:** 2019-06-15

**Authors:** A. Docampo-Seara, M. Lanoizelet, R. Lagadec, S. Mazan, E. Candal, M. A. Rodríguez

**Affiliations:** 1 Departamento de Bioloxía Funcional, Centro de Investigación en Bioloxía (CIBUS), Universidade de Santiago de Compostela, 15782 Santiago de Compostela, Spain; 2 CNRS, Sorbonne Universités, UPMC Univ Paris 06, UMR7232, Observatoire Océanologique, Banyuls sur Mer, France

**Keywords:** Olfactory bulb, Catshark, Mitral cells, Development, Glutamatergic lineage, Pax6

## Abstract

In mammals, the development of the olfactory bulb (OB) relies in part on the expression of transcription factors involved in the specifications/differentiation of glutamatergic cells. In a previous study from our group, a high molecular similarity was reported between mammals and cartilaginous fishes regarding the neurogenic mechanisms underlying the development of glutamatergic cells in the telencephalon. However, information about the transcriptional program operating in the development of the glutamatergic system (mainly represented by mitral cells) in the OB is lacking in the catshark *Scyliorhinus canicula*, a cartilaginous fish. Using immunohistochemistry and in situ hybridization techniques, we have found that, previously to the appearance of the olfactory primordium (OP), proliferating cells expressing Pax6 with molecular hallmarks of progenitor radial glia were located in the ventrolateral pallial ventricular zone. Later in development, when the OP is recognizable, a stream of Pax6-positive cells were observed between the ventricular zone and the OP, where transcription factors involved in mitral cell development in mammals (*ScTbr2*, *ScNeuroD,* Tbr1) are expressed. Later in development, these transcription factors became expressed in a layered-like structure where *ScVglut1*, a marker of mitral cells, is also present. Our data suggest that the transcriptional program related with the specification/differentiation of glutamatergic cells in the telencephalon has been conserved throughout the evolution of vertebrates. These results, in combination with previous studies concerning GABAergic neurogenesis in sharks, have evidenced that the OB of mammals and sharks shares similarities in the timing and molecular programs of development.

## Introduction

The sense of smell is essential for a variety of behaviors in vertebrates like mating, feeding, fear and aggression. The organization of the olfactory system is well conserved in vertebrates not only in terms of function, but also in connectivity and also concerning the developmental origin of different structures within the peripheral and central olfactory system. Numerous investigations in mammals indicate that the olfactory system constitutes an excellent model to study various developmental aspects of the nervous system such as neurogenesis, neuronal migration, and axon guidance (Blanchart et al. 2011; Díaz-Guerra et al. 2013; Lim and Alvarez-Buylla 2016).

In rodents, the main olfactory system is related with the olfactory chemoreception of odorants and comprises a primary olfactory pathway consisting of the nasal olfactory epithelium (OE) and the main olfactory bulb (MOB), and a secondary olfactory pathway that includes all cortical regions directly innervated by MOB projection neurons (known together as the olfactory cortex); the olfactory cortex in turn releases signals to higher cortical areas involved in conscious perception and to limbic areas that control basic drives and emotions (reviewed in Boehm 2006; Treloar et al. 2010).

Besides, olfactory chemoreception of pheromones depends on an accessory olfactory system called vomeronasal system (VNS) that comprises the neuroepithelium of the vomeronasal organ (VNO or Jacobson’s organ) and the accessory olfactory bulb (AOB); signals from the AOB are relayed to regions of the amygdala and hypothalamus implicated in behavioral and physiological effects of pheromones (for review see Boehm 2006; Huilgol and Tole 2016).

In rodents, projecting axons of the olfactory receptor neurons located in the OE reach the telencephalic vesicle and induce the growth of the olfactory bulb primordium (OP). The MOB becomes evident macroscopically around the day 12 of embryonic development (E12; Gong and Shipley 1995). During the morphogenesis of the mouse MOB, projection neurons (mitral cells) are born from pallial progenitor cells and, then, interneurons (granule and periglomerular cells) that arise  from the subpallium migrate tangentially toward their destination within the MOB (Blanchart et al. 2006; Vergaño-Vera et al. 2006; Imamura and Greer 2013; Huilgol and Tole 2016). The cellular organization of the MOB and AOB is similar. Projection neurons of the MOB and anterior AOB arise from the same region; however, differences in the domains of origin and migration routes of projection neurons are present between the anterior and posterior regions of the AOB. Interneurons of the MOB and AOB are born in the same region (for review see: Huilgol et al. 2013; Huilgol and Tole 2016).

Despite most studies about the olfactory system are focused on mammals, other animal models are necessary nowadays to understand different embryological aspects occurring during development of the olfactory system. Cartilaginous fishes represent one of the three living lineages of vertebrates (cyclostomes, cartilaginous fishes and bony vertebrates). Cartilaginous fishes diverged from bony vertebrates about 450 million years ago. Embryological studies in cartilaginous fishes reveal a conserved pattern of gene expression in diverse developmental process across gnathostome vertebrates (Gillis and Shubin 2009), and also morphogenetic processes and regionalization patterns are strikingly similar to mammals (Rodríguez-Moldes et al. 2017). More recently, the whole-genome analysis of three elasmobranch species has shown the presence of genes related with homeostasis, reproduction, and mechanisms for the generation of neuronal cell diversity homologous to that found in mammals (Hara et al. 2018). These studies indicate that cartilaginous fishes represent a key model for better knowledge of evolution of gnathostome brain development.

Moreover, cartilaginous fishes possess a well-developed sense of smell that is important for survival, localizing preys, avoiding predators, and chemosensory communication (for review see: Yopak et al. 2015). Numerous investigations have been referred to the adult olfactory system in elasmobranchs fishes. Several studies have been referred to the cell organization of the OE of the catshark, where sensory ciliated neurons, which in tetrapod vertebrates project to the MOB, are lacking; however, in the OE, microvillous olfactory receptor neurons and crypt sensory neurons are clearly recognizable (Theisen et al. 1986; Ferrando et al. 2006a, b, 2009, 2010, 2012; Zaccone et al. 2011). In addition, genomic studies show that the predominant olfactory receptor type in the catshark and the elephant shark is the vomeronasal type2 receptor (V2R) (Sharma et al. 2019). These evidences together with ultrastructural and immunohistochemical data indicate that in the catshark the  olfaction could mainly rely on a VNS (Ferrando and Gallus 2013). Interestingly, anatomical and molecular data show a primordial accessory olfactory system in the sea lamprey (Chang et al. 2013) and an accessory olfactory system was also identified in the African lungfish (González et al. 2010) and zebrafish (Biechl et al. 2017).

Though the OBs are laminated structures, in sharks, they do not present the six cell layers described in mammals. The cytoarchitectonic organization of the OB in the catshark has been investigated by classic staining techniques and summarized by Smeets et al. (1983). Three main layers can be observed in this species: the olfactory nerve layer, the glomerular layer and the granular layer. Two main types of cells have been described in the OB: interneurons and projection neurons. The ultrastructure of the OB (Dryer and Graziadei 1996) and the arrangement of the primary and secondary olfactory projections have been described in different elasmobranchs species (Dryer and Graziadei 1993, 1994; Yáñez et al. 2011). Besides, in juveniles and/or adults of *S. canicula,* the OB have been characterized using antibodies against enzymes like tyrosine hydroxylase (TH) (Carrera et al. 2012), glutamate acid decarboxylase (GAD) (Sueiro 2003), neuronal nitric oxide synthase (nNOS) (Ferrando et al. 2012) and choline acetyltransferase (ChAT) (Anadón et al. 2000); other neuroactive substances such as glycine (Anadón et al. 2013), serotonin (Carrera et al. 2008a) and diverse neuropeptides (Rodríguez-Moldes et al. 1993; Molist et al. 1995; Teijido et al. 2002) have also been detected in the OB of the catshark.

In contrast, studies about development of the olfactory system are scarce and mainly focused on the peripheral olfactory system (Ferrando et al. 2012; Ferreiro-Galve et al. 2012; Quintana-Urzainqui et al. 2014). Using tract-tracing and immunohistochemical techniques, the development of the peripheral olfactory system of the catshark *Scyliorhinus canicula* has been described, and numerous Pax6-expressing cells have been observed in the OE, and along the developing olfactory nerve (Ferreiro-Galve et al. 2012; Quintana-Urzainqui et al. 2014). In addition, numerous Pax6-positive cells have been also reported in the ventricular zone of the ventrolateral pallium of embryos before the appearance of the OP (Ferreiro-Galve et al. 2012). However, the phenotype of these cells is unknown and their relationship with OB development has not been addressed so far. On the other hand, information about the origin, specification and differentiation of the OB cell types in the catshark is scarce and restricted to dopaminergic cells. These cells originate in a subpallial ventricular domain in late embryos and reach the OB following a route named lateral stream. The presence of TH-ir cells in this stream, as well as in the OB of stage 32 embryos, indicate that these cells may be the source of the granular and periglomerular cells of the mature OB (Ferreiro-Galve et al. 2012; Carrera et al. 2012; Quintana-Urzainqui et al. 2015). However, information about the development of OB glutamatergic cells (mitral cells) is lacking in sharks.

The transcriptional program involved in the specification and differentiation of mitral cells is well known in mammals and, curiously, is the same that operates on the specification/differentiation of pallial glutamatergic neurons before the mature organization of the OB is achieved (for review see: Bulfone et al. 1998; Englund et al. 2005; Díaz-Guerra et al. 2013; Imamura and Greer 2013; Kahoud et al. 2014; Roybon et al. 2015; Mihalas and Hevner 2017). Different experimental approaches carried out in mammals have shown that the transcription factor-paired homeobox 6 (Pax6), together with Sox2 (a transcription factor expressed in stem cells), is involved in neural stem cell self-renewal, neurogenesis and differentiation of specific neural cell types (Dellovade et al. 1998; Nomura and Osumi 2004; Kohwi et al. 2005; Sansom et al. 2009; Gómez-López et al. 2011; Curto et al. 2014). Pax6-expressing cells have been reported in the ventricular zone of the embryonic pallium as well as in the developing OB in mammals (Stoykova and Gruss 1994; Puelles et al. 2000), amphibians (Franco et al. 2001) and elasmobranch fishes, even though in the catshark, Pax6 is expressed throughout the VZ of the pallium, as in other vertebrates, the gradient described in mammals (lateral high, medial low and anterior high, and posterior low) is not evident in sharks (Ferreiro-Galve et al. 2012; Quintana-Urzainqui et al. 2012; Rodríguez-Moldes et al. 2017). In the MOB of rodents, projection neurons (mitral and tufted cells) derive from Pax6-positive radial glia progenitors located in the ventricular zone of the dorsal pallium (Winpenny et al. 2011; Imamura et al. 2011; Imamura and Greer 2013). Besides, Pax6 regulates the expression of two T-box genes (Tbr1 and Tbr2, the latter also known as Eomes) (Bulfone et al. 1995; Méndez-Gómez et al. 2011; Mizuguchi et al. 2012; Imamura and Greer 2013), which are also expressed along mitral cell development in the OB in rodents (Bulfone et al. 1999; Faedo et al. 2002; Winpenny et al. 2011; Mizuguchi et al. 2012; Roybon et al. 2015), birds (Bulfone et al. 1999), amphibians (Moreno et al. 2003; Brox et al. 2004) and zebrafish (Mione et al. 2001; Mueller and Wulliman 2016). In addition, the basic helix–loop–helix transcription factor NeuroD is also implicated in the terminal differentiation of mitral cells (Boutin et al. 2010; Osorio et al. 2010; Roybon et al. 2015). At the end of the embryonic period in mammals, mitral cells begin to express the glutamate vesicular transporter 1 (Vglut1; Ohmomo et al. 2011), which is also a marker of mitral cells in other groups of vertebrates such as reptiles (Sarkar and Atoji 2018). Mutant mice, where expression of Pax6, Tbr1 and Tbr2 is altered, show a disrupted OB morphogenesis indicating that this transcriptional cascade plays a key role in the correct morphogenesis of the OB (Bulfone et al. 1998; Nomura and Osumi 2004; Kahoud et al. 2014). Curiously, in mammals Tbr1 is also implicated in the specification of the anterior AOB; however, the specification of the posterior AOB is under the control of different genes (for review see: Huilgol and Tole 2016).

With the purpose of shedding light into the development of the central olfactory system in an evo–devo context, we have characterized Pax6 immunoreactive cells present in the ventricular zone of the ventrolateral pallium embryos of catshark with different progenitor markers such as Sox2 (stem cells), GFAP and BLBP (radial glia cells) and PCNA (proliferating cells). Then, we have studied the expression pattern of Pax6, once the OP emerges, and we have analyzed the expression pattern of transcription factors (such as Tbr2, NeuroD and Tbr1) and the vesicular transporter of glutamate 1 (vGlut1) related to differentiation of glutamatergic cells in the developing OB using immunocytochemistry and in situ hybridization techniques. In addition, we have carried out a BrdU pulse-chase study to determine the developmental period where these cells are generated. Finally, we have discussed our results in an evo–devo context and also at the light of the possibility of the existence of an AOB in the catshark.

## Materials and methods

### Experimental animals

In the present study, we have analyzed 15 embryos of *S. canicula* from stages 30 (S30) to 32 (S32) of development. Embryos were provided by the Marine Biological Model Supply Service of the CNRS UPMC Roscoff Biological Station (France) and the Oceanographic Observatory of Banyuls sur Mer (France). Embryos were staged by their external features according to Ballard et al. (1993). Sharks were raised in seawater tanks under standard conditions of temperature (15–16 °C), pH (7.5–8.5) and salinity (35 g/L) and suitable measures were taken to minimize animal pain and discomfort. All procedures were made according to the guidelines established by the European Communities Council Directive of 22 September 2010 (2010/63/UE) and by Spanish Royal Decree 53/2013 for animal experimentation and were approved by the Ethics Committee of the University of Santiago de Compostela.

### Tissue processing

Embryos were deeply anesthetized with 0.5% tricaine methane sulfonate (MS-222; Sigma, St. Louis, MO, USA) in seawater and separated from the yolk before fixation in 4% paraformaldehyde (PFA) in elasmobranch’s phosphate buffer [EPB: 0.1 M phosphate buffer (PB) containing 1.75% of urea, pH 7.4] for 48–72 h depending on the stage of development. Subsequently, they were rinsed in PB saline (PBS), cryoprotected with 30% sucrose in PBS, embedded in OCT compound (Tissue Tek, Torrance, CA), and frozen with liquid nitrogen-cooled isopentane. Parallel series of sections (16–18-μm thick) were obtained in transverse planes on a cryostat and mounted on Superfrost Plus (Menzel-Glasser, Madison, WI, USA) slides.

### BrdU pulse-chase experiments

BrdU pulse-chase labeling was performed by incubating three catshark embryos at S28, S29 and S30 in 10 mg/ml of BrdU in oxygenated artificial sea water, after opening the egg shell, for 2 h 30 min; embryos were then moved to untreated sea water until they achieve S31, when they were killed by overdose of MS-222 and fixed by immersion in PFA 4% for 48 h. For detection of BrdU, sections were incubated in 2 N HCl for 30 min at 50 °C to denature DNA strands. HCl reaction was stopped by addition of 0.1 M sodium tetraborate and sections were then rinsed in TBS for 10 min before antibody incubation. Sections were incubated with anti-BrdU antibody at room temperature (RT) overnight and processed for immunofluorescence as described below.

### In situ hybridization

We applied in situ hybridization (ISH) for *S. canicula Tbr2/Eomes, NeuroD1, vGlut1 and Sox2 (ScSox2, ScTbr2, ScNeuroD1, ScvGlut1)* genes. These probes were selected from a collection of *S. canicula* embryonic cDNA library (mixed stages S9–S22), submitted to high-throughput EST sequencing (coordinated by Dr. Sylvie Mazan). Sense and antisense digoxigenin-UTP-labeled *ScTbr2, ScNeuroD1, ScvGlut1 and ScSox2* were synthesized directly by transcription in vitro. ISH was performed on cryostat sections of S30, S31 and S32 embryos following standard protocols (Coolen et al. 2009). Briefly, sections were permeabilized with proteinase K, hybridized with sense or antisense probes overnight at 65 °C and incubated with the alkaline phosphatase-coupled anti-digoxigenin antibody (1:2000, Roche Applied Science, Manheim, Germany) overnight at 4 °C. The color reaction was performed in the presence of BM-Purple (Roche). Color reaction was stopped by rinsing in PFA 4% for 45 min. Finally, sections were dehydrated and coverslipped. Control sense probes did not produce any detectable signal.

### Immunohistochemistry

Sections were pre-treated with 0.01 M citrate buffer pH 6.0 for 30 min at 90 °C for heat-induced epitope retrieval and allowed to cool for 20 min at RT. Sections were rinsed in 0.05 M Tris-buffered saline (TBS) pH 7.4 for 5 min and treated with 10% H_2_O_2_ in TBS for 30 min at RT to block endogenous peroxidase activity. Sections were rinsed in 0.05 M TBS pH 7.4 for 5 min and incubated approximately for 15 h at RT with primary antibodies (see Table 1). Sections were rinsed three times in 0.05 M TBS pH 7.4 for 10 min each, and incubated in the appropriate HRP-coupled secondary antibody (see Table 1) for 1 h at RT. All dilutions were made with TBS containing 15% normal goat serum (Millipore, Billerica, MA, USA) 0.2% Triton X-100 (Sigma) and 2% bovine serum albumin (BSA, Sigma). All incubations were carried out in a humid chamber. Then, sections were rinsed three times in 0.05 M TBS pH 7.4 for 10 min each. The immunoreaction was developed with 0.25 mg/ml diaminobenzidine (DAB) tetrahydrochloride (Sigma) in TBS pH 7.4 and 0.00075% H_2_O_2_, or with SIGMAFAST™ 3.3-DAB tablets as indicated by the manufacturers. In Pax6 samples, 2.5 mg/ml nickel ammonium sulfate was added. Finally, the sections were dehydrated, and coverslipped.

**Table 1:** Primary and secondary antibodies used

Primary antibody	Source	Working dilution	Secondary antibody	Source	Working dilution
Pax6	Policlonal rabbit anti-Pax6 Covance (Cat. No. PRB-278P)	1:300	Goat anti-rabbit HRP coupled	Dako, Glostrup, Denmark	1:200
PCNA	Monoclonal mouse anti-PCNA Sigma (Cat. No. P8825)	1:500	488-cojugated donkey anti-mouse	Alexa Fluor Molecular Probes, Eugene, OR	1:200
GFAP	Polyclonal rabbit anti-GFAP Dako (Cat. No. Z033429)	1:500	546-conjugated donkey anti-rabbit	Alexa fluor Molecular Probes, Eugene, OR	1:200
BLBP	Polyclonal rabbit anti-BLBP Millipore (Cat. No. ABN14)	1:300	FITC-conjugated goat anti-rat	ThermoFisher Cat. No. 31621	1:100
Tbr1	Policlonal rabbit anti-Tbr1 Chemicon (Cat. No. AB9616) Millipore (Cat. No. AB10554)	1:200			
TH	Monoclonal mouse anti-TH Millipore (Cat. No. MAB318)	1:500			
BrdU	Policlonal Rat anti-Brdu Abcam (Cat. No. ab6326)	1:100			

### Double in situ hybridization–immunohistochemistry

We applied double in situ hybridization–immunohistochemistry for *ScTbr2* and *ScNeuroD* probes and the Tbr1 antibody. After colorimetric detection of probes, sections were rinsed three times in 1 M PBS for 10 min each, rinsed in PFA 4% for 45 min and immunohistochemistry was performed as described above.

### Double immunofluorescence

For heat-induced epitope retrieval, sections were pre-treated with 0.01 M citrate buffer (pH 6.0) for 30 min at 90 °C and allowed to cool for 20 min at RT. Sections were rinsed in 0.05 M TBS (pH 7.4) for 5 min and incubated approximately for 15 h at RT with primary antibodies (see Table 1). Sections were rinsed three times in 0.05 M TBS pH 7.4 for 10 min each, and incubated in the appropriate combination of fluorescent dye-labeled secondary antibodies (see Table 1) for 1 h at RT. All dilutions were made with TBS containing 15% normal donkey serum (Millipore, Billerica, MA, USA) 0.2% Triton X-100 (Sigma) and 2% bovine serum albumin (BSA, Sigma). All incubations were carried out in a humid chamber. Sections were rinsed three times in 0.05 M TBS pH 7.4 for 10 min each and in distilled water for 30 min. Sections were then allowed to dry for 30 min at 37 °C, and mounted in MOWIOL 4-88 Reagent (Calbiochem, MerkKGaA, Darmstadt, Germany). Information about the primary and secondary antibodies is included in Table 1.

### Control and specificity of antibodies

The PCNA antibody has been previously used to label progenitor cells in the brain and olfactory system of *S. canicula* (i.e. Quintana-Urzainqui et al. 2014, 2015). In addition, the specificity of the antibody against Pax6 has been tested by pre-absorption test in *S. canicula* (Ferreiro-Galve et al. 2012). The Tbr1 antibody has been previously used as a marker of pallial glutamatergic neurons in the developing brain of *S. canicula* (Docampo-Seara et al. 2018a). Antibodies against glial markers GFAP and BLBP and the enzyme TH (marker of dopaminergic cells) have been previously tested by western blot (Carrera et al. 2012; Docampo-Seara et al. 2018b).

### Imaging

Fluorescent sections were photographed with the Leica TCS-SP2 scanning microscope with a combination of blue and green excitation lasers. Confocal images were acquired separately for each laser channel with steps of 2 μm along the z-axis, and collapsed images were obtained with the LITE software (Leica). Light field images were obtained with an Olympus BX51 microscope equipped with an Olympus DP71 color digital camera. Images were adjusted for contrast, brightness and intensity using Corel Draw X7.

## Results

In the catshark, the OP emerges as a well-defined protrusion in ventrolateral portions of the telencephalic hemispheres in the transition from S30 to S31 of development (Ferreiro-Galve et al. 2012). While no cell layering can be appreciated at this stage, incipient glomeruli began to appear, called protoglomeruli, in the distal portion of the OP. At S32, protoglomeruli are more evident and the OB becomes conspicuous and organized in three basic layers, which are, from outside to inside, the olfactory nerve layer, the glomerular layer (protoglomeruli) and the granular layer. Mitral cells, which in the mammalian brain and in other vertebrates constitute a proper layer, do not form a defined layer in the catshark, but rather they are diffusely distributed between the glomerular and granular layers. From S32 onwards, the OB grows and the glomerular layer and granular layer form well-defined layers. For further information about the OB development in the catshark, see Quintana-Urzainqui et al. (2014, 2015).

### Expression of Pax6 and progenitor cell markers in the ventrolateral pallium at stage 30

**Fig. 1:**
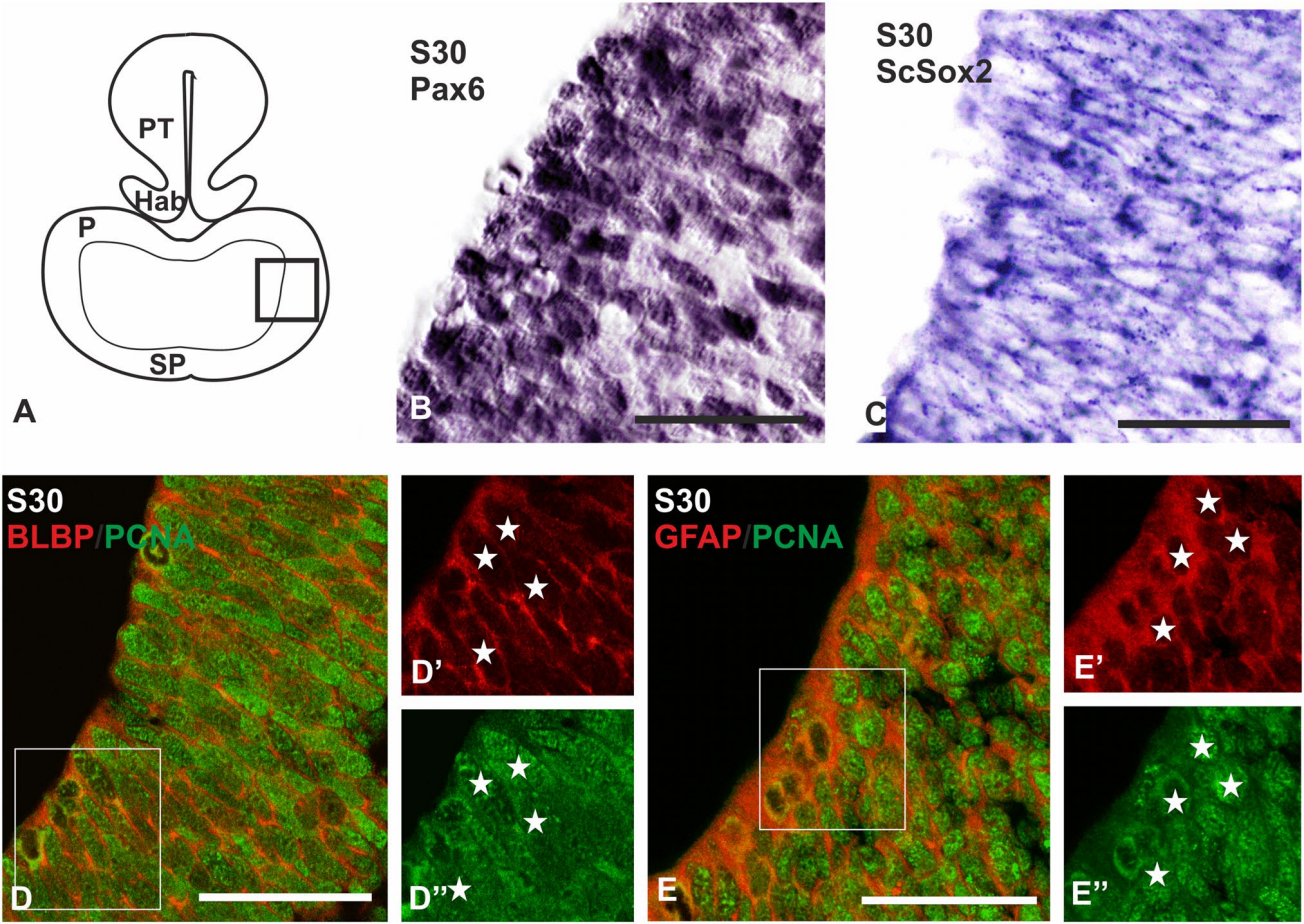
Transverse sections showing the expression pattern of progenitor markers in the ventricular zone of the telencephalic walls of S30 of development. **a** Scheme showing the main divisions of the developing brain of a S30 of development. The square represents the portion of the pallium studied at this stage of development. **b** Photomicrograph at high magnification showing the expression pattern of Pax6 in the ventricular lateral portions of the pallium. Note two different intensities of immunolabeling. **c** Photomicrograph at high magnification showing the expression pattern of *ScSox2*. **d**, **d’’** Double immunofluorescence BLBP/PCNA in the lateral portions of the pallium showing double-labeled cells (stars). **e**, **e’’** Double immunofluorescence GFAP/PCNA in the lateral portions of the pallium showing double-labeled cells (stars). Scale bars: 25 µm. *P* pallium, *Hab* habenula, *PT* pretectum, *SP* subpallium

At S30 of development, before the appearance of the OP, the telencephalon is constituted by a large ventricle surrounded by the telencephalic walls. These walls are constituted by a proliferating ventricular zone and an intermediate zone comprised of neuroblasts (Docampo-Seara et al. 2018a). Pallium and subpallium are recognizable because of their differential expression of Pax6 and GAD, respectively (Carrera et al. 2008b; Quintana-Urzainqui et al. 2015; Rodríguez-Moldes et al. 2017), but their main pallial and subpallial subdivisions are not yet established (Fig. 1a). As mitral cell progenitors could be present in the pallial proliferating ventricular zone before the appearance of the OP in a way similar to what happens with mitral cell progenitors in the OB of mammals (Imamura et al. 2011), we have studied the expression pattern of Pax6 (pallial/mitral progenitor marker), *ScSox2* (stem cell marker), BLBP–PCNA and GFAP–PCNA (radial glial progenitor cell markers) in the ventricular zone of the ventrolateral portions of the pallium (the presumptive area where the OP is going to emerge) of a S30 embryo (square in Fig. 1a).

Pax6 expression in the pallium was restricted to the pallial ventricular zone in the telencephalon of a S30 embryo, including ventrolateral portions (Fig. 1b). *ScSox2* expression was also present in the pallial ventricular zone, where its expression coincides with that of Pax6 (compare Fig. 1b and c). In the same region where numerous Sox2- and Pax6-positive cells were present, numerous BLBP- and GFAP-positive cells were observed; immunoreactivity for both radial glia markers was present in the periphery of the cell bodies and in their basal and apical processes. Double immunofluorescence BLBP/PCNA and GFAP/PCNA showed that all BLBP- and GFAP-expressing cells also expressed PCNA (Fig. 1d, and stars in Fig. 1d’, d’’; Fig. 1e, and stars in Fig. 1e’, e’’).

### Expression pattern of transcription factors (Pax6, *ScTbr2*, Tbr1 and *ScNeuroD*) in S31/S32 embryos

**Fig. 2:**
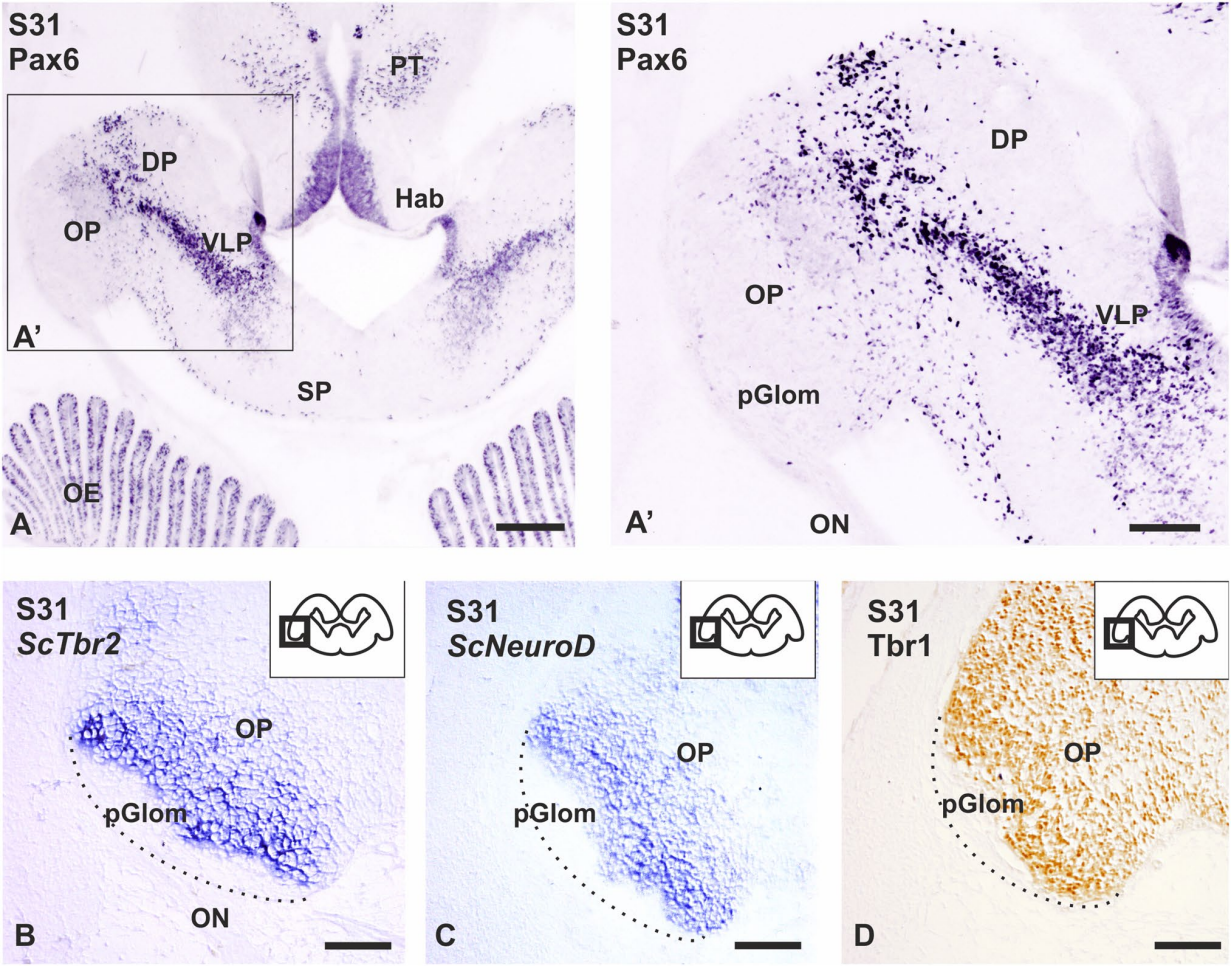
Transverse sections showing the expression pattern of Pax6 and the neurogenic markers *ScTbr2, ScNeuroD* and Tbr1 in the OP of S31 of development. **a** Photomicrograph at low magnification of the telencephalon showing the expression pattern of Pax6 in a S31 of development. **a’** Detail of the expression pattern of Pax6 showing a stream of Pax6-positive cells coursing from the ventricular zone to the OP. Note that Pax6-positive cells in the OP show low levels of Pax6. In S31, *ScTbr2* (**b**) and *ScNeuroD* (**c**) are expressed as a specific cell band close to the prospective glomerular layer, meanwhile Tbr1 is expressed in all regions of the OP (**d**). Dotted lines show the limit of the OP. Scale bars: 200 µm (**a**), 100 (**a**’–**d**). *DP* dorsal pallium, *Hab* habenula, *OE* olfactory epithelium, *ON* olfactory nerve, *OP* olfactory primordium, *PT* pretectum, *SP* subpallium, *VLP* ventrolateral pallium

We have studied the main transcription factors involved in mitral cell development (Pax6, Tbr2, NeuroD and Tbr1) at S31 of development (after the OP emerges) and at S32 (when the basic cytoarchitectonic pattern of the OB begins to appear). At S31, numerous Pax6 immunoreactive cells can be observed in the ventricular zone of dorsal and ventrolateral pallial regions (Fig. 2a). Interestingly, a stream of Pax6-positive cells can be appreciated coursing from the ventricular zone of the ventrolateral pallium to the OP (Fig. 2a’). Curiously, cells that seem to invade the OP show a weaker Pax6 immunoreactivity. Besides, we have also analyzed the expression pattern of *ScTbr2*, *ScNeuroD*, and Tbr1. Intense *ScTbr2* and *ScNeuroD* labeling was detected in a cell band adjacent to the most distal region of the OP, where the first protoglomeruli appear, close to the ON entry (Fig. 2b, c), while a faint labeling was observed in proximal regions of the OP. Note that the band of intense labeled cells of both *ScTbr2* and *ScNeuroD* fits with the Pax6-negative territory of the OP (compare Fig. 2a with Fig. 2b and c). In contrast, Tbr1 immunohistochemistry revealed numerous immunoreactive cells in all regions of the OP, except in the region of protoglomeruli (Fig. 2d).

**Fig. 3:**
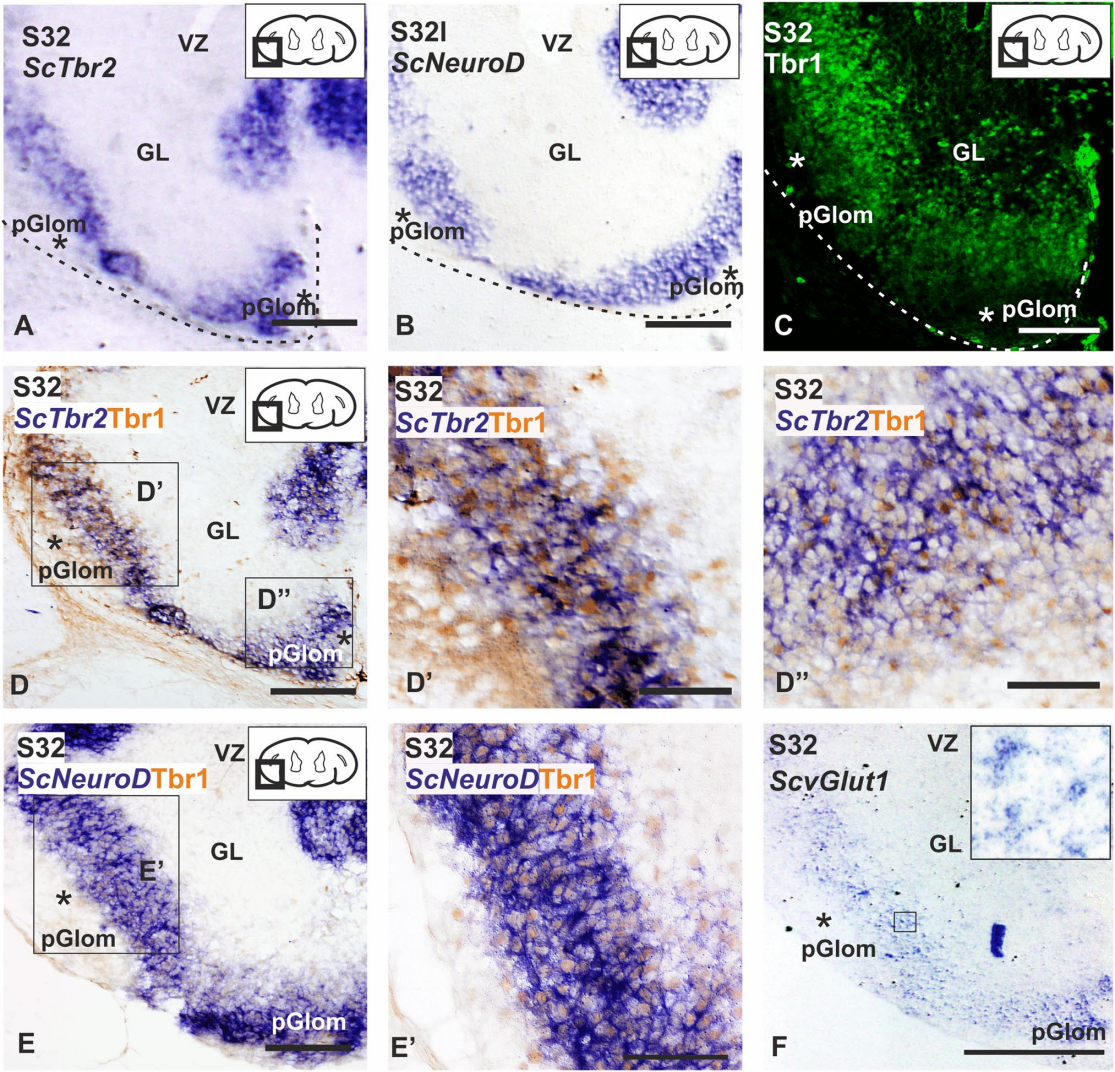
Transverse sections showing the expression pattern of the neurogenic markers *ScTbr2, ScNeuroD*, Tbr1 and *ScvGlut1* in the OP of S32 of development. In S32 embryos *ScTbr2* (**a**), *ScNeuroD* (**b**) and Tbr1 (**c**) show a similar expression pattern. Note that Tbr1 seems to label a wider territory compared with *ScTbr2* and *ScNeuroD*. Dotted lines shows the limit of the OB. Asterisks show protoglomeruli. Double ISH–IHC between *ScTbr2* and Tbr1 (**d**) in the lateral (**d**’) and ventral (**d**’’) portions of the distal OB, showing double-labeled cells. Note that in the lateral portion Tbr1 immunoreactivity is more abundant (**d**’), in contrast to the ventral portion, where *ScTbr2* is more abundant (**d**’’). **e**, **e**’ Double ISH–IHC between *ScNeuroD* and Tbr1 showing an almost total colocalization of both neurogenic markers. **f** Photomicrograph showing the expression pattern of *ScvGlu*t1, coincident with the expression of *ScTbr2, ScNeuroD* and Tbr1. Scale bars: 100 µm (**a**, **b**, **c**, **d**, **e**, **f**), 50 µm (**d**’, **d**’’, **e**’). *GL* granular layer, *OP* olfactory primordium, *pGlom* protoglomeruli, *VZ* ventricular zone

At S32, the expression pattern of the three transcription factors was restricted to a dense layer of cells in the region adjacent to the protoglomeruli (Fig. 3a–c). As in S31, Tbr1-positive cells were also located in proximal regions of the prospective OB (Fig. 3c). Since the expression pattern of *ScTbr2* and *ScNeuroD* was highly coincident but the territory of Tbr1 expression seems to be wider, we performed double in situ hybridization–immunohistochemistry for *ScTbr2* and Tbr1 and for *ScNeuroD* and Tbr1 at S32 (Fig. 3d, e’). We found that most of the *ScTbr2*-expressing cells were also positive for Tbr1 (Figs. 3d, d’). However, some cells expressed only *ScTbr2* or Tbr1. Curiously, most cells that only expressed Tbr1 were located in lateral regions of the distal OB (Fig. 3d’), in contrast to the ventral region, where most of the cells were positive for Sc*Tbr2* only (Fig. 3d’’). Concerning *ScNeuroD* and Tbr1 cell populations, we have observed a high coexpression of both factors in the same cells (Fig. 3e, e’) and no differences between the lateral and ventral regions of the OB were appreciated.

In mammals and other vertebrate groups, these transcription factors are part of a transcriptional code related with the differentiation of pallial glutamatergic neurons (reviewed by Hevner et al. 2006). However, there is no direct evidence about the involvement of these factors in the glutamatergic system in sharks. We have analyzed the expression of the glutamatergic marker *ScvGlut1* (vesicular glutamatergic transporter) in S32 embryos by in situ hybridization. We found numerous *ScvGlut1*-positive cells adjacent to the protoglomerular region (Fig. 3f), in the region occupied by *ScTbr2*/Tbr1/*ScNeuroD*-expressing cells. In addition, we have performed double immunofluorescence in early S32 embryos for Tbr1 and the enzyme tyrosine hydroxylase (TH), which is involved in dopamine synthesis and labels interneurons in the OB of sharks (Quintana-Urzainqui et al. 2015); Tbr1-expressing cells were observed at this developmental stage; however, TH immunoreactivity was still not observed in the OB (data not shown).

### Expression pattern of BrdU in mitral cells presumptive territory at S31 after pulses at different developmental stages

**Fig. 4:**
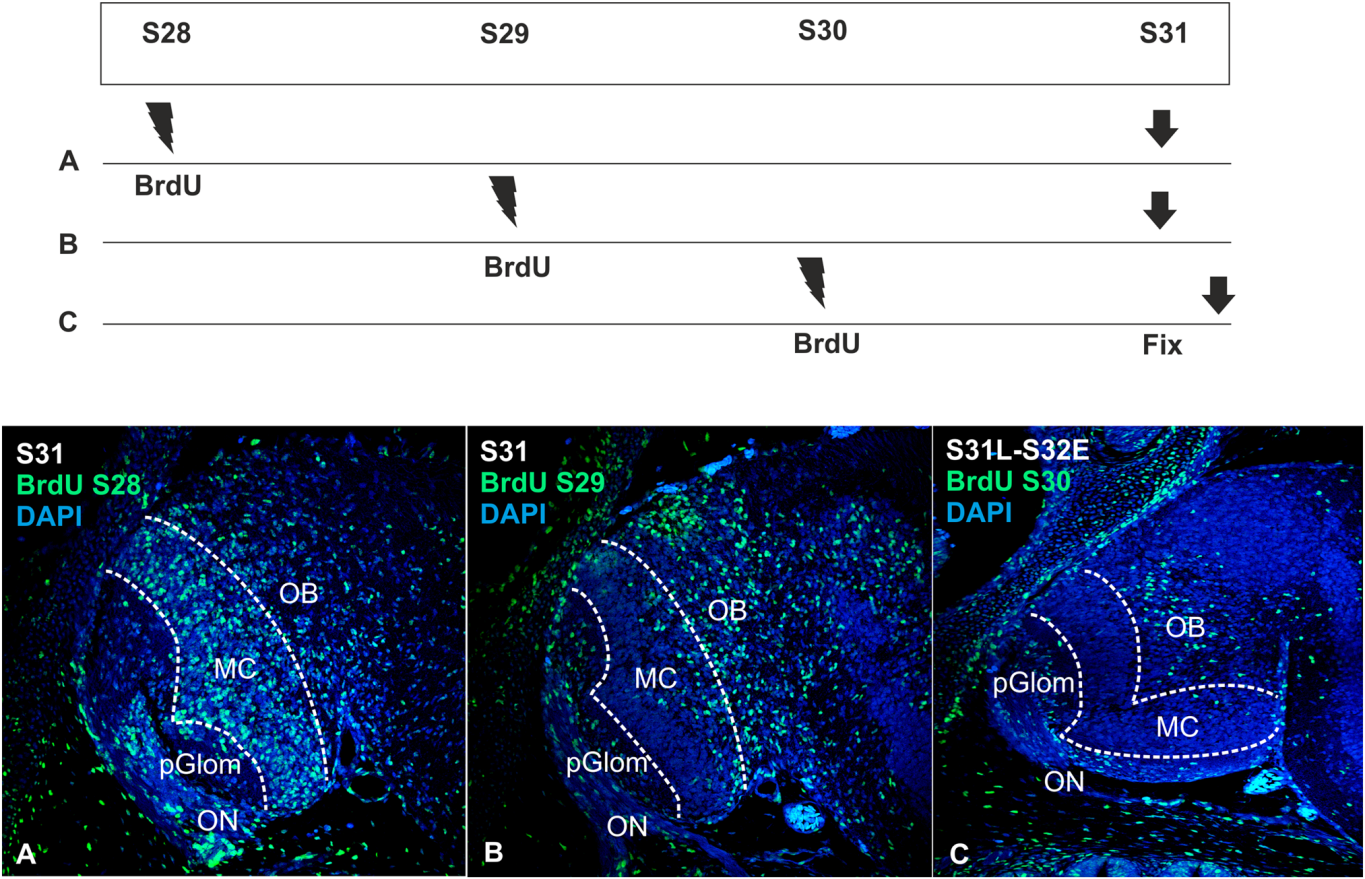
Scheme and transverse sections showing BrdU immunoreactive cells (green), counterstained with DAPI (blue), in the OB of a S31 embryo after BrdU pulses at S28 (**a**), S29 (**b**) and S30 (**c**). Note that most BrdU-positive cells in mitral cell territory belong to pulses in S28 (**a**), in contrast to S30 (**c**), where almost none positive cells can be detected. Dotted lines represent *ScTbr2-* and *ScNeuroD*-positive territories, where mitral cells are located. *MC* mitral cell territory, *OB* olfactory bulb, *ON* olfactory nerve, *pGlom* protoglomeruli

As we have determined that cells in the ventrolateral VZ of the pallium typically express mitral cell progenitor markers before the emergence of the OP, we have decided to determine at which point of development, mitral cells are generated. For that, BrdU pulse experiments were performed in catshark embryos at S28, S29 and S30 and embryos were allowed to develop up to S31. When pulses were performed at S28, most BrdU-positive cells at S31 were concentrated in the portion of the OP that corresponds to the territory labeled by *ScTbr2* (Fig. 4a, dotted lines correspond to *ScTbr2* territory, see Fig. 2c). On the other hand, when pulses were performed at S29, most BrdU-positive cells at S31 were concentrated in the prospective granular layer, with a small proportion of positive cells in the olfactory region that would correspond to mitral cells location (Fig. 4b). Finally, when pulses were carried out at S30, barely no BrdU-positive cells were found in the presumptive location of mitral cells at S31; in contrast, the vast majority of cells were located at the granular portion of the OP (Fig. 4c).

## Discussion

In the present work, we have studied the phenotype of Pax6-expressing cells observed in the VZ of the lateroventral pallium of catshark embryos in terms of expression of proliferation and radial glial markers. In addition, we have analyzed the expression of several transcription factors implicated in the differentiation of glutamatergic lineage in the primordial OB of embryos (Pax6, *ScTbr2, ScNeuroD*, Tbr1). Our results suggest that mitral cells originate from Pax6-expressing radial glial cells in the VZ of the ventrolateral portion of the pallium that express the same transcription factors described for mitral cell progenitors in mammals (see “Discussion” below).

### Pax6-positive cells present in the embryonic telencephalic ventricular zone are proliferating stem cells with molecular hallmarks of radial glia

Several investigations in mammals have shown that Pax6 is involved in the development of the olfactory placode, the OB and the olfactory cortex (Stoykova and Gruss 1994; Nomura et al. 2007). In amphibians, the expression pattern of Pax6 in the developing OB suggests that this transcription factor also plays a key role in olfactory system development (Franco et al. 2001; Moreno et al. 2008; Joven et al. 2013). In the embryonic OP of mammals, this transcription factor is highly expressed in the ventricular zone (Puelles et al. 2000; Vergaño-Vera et al. 2006). Using molecular markers of radial glia, it was shown that Pax6 is localized in progenitor radial glial cells during mammalian pallial neurogenesis, including the ventricular zone of the embryonic OB (Götz et al. 1998; Imamura and Greer 2013). In addition, mouse neural stem cell lines display many hallmarks of radial glia, like bipolar morphology, including BLBP and Pax6 expression (Conti et al. 2005; Pollard et al. 2006). The level of Pax6 is directly related with neural stem cell self-renewal, proliferation and differentiation (Sansom et al. 2009; Gómez-López et al. 2011).

Previous studies in catshark embryos show that Pax6 is expressed in the developing OE, and immature neurons positive for Pax6 are present along olfactory axons, as it happens in other vertebrates (see Ferreiro-Galve et al. 2012; Quintana-Urzainqui et al. 2014 and references therein). In addition, previous to the emergence of the OP (S30), numerous Pax6 cells were observed in the ventricular zone of the ventrolateral pallium (present results; Ferreiro-Galve et al. 2012). As the molecular phenotype of these cells was not addressed, we have compared the expression pattern of Pax6 with the expression pattern of stem cell marker (*ScSox*2) and glial markers (GFAP and BLBP) in the embryonic S30 of catshark.

In S30 embryos, numerous Pax6*-*expressing cells are present in the ventrolateral ventricular zone of the pallium, and differences in the intensity of the Pax6 labeling between positive cells close to the ventricle and cells located far from the ventricle are observed, which are in agreement with the previous studies in the catshark (Ferreiro-Galve et al. 2012). *ScSox2*-expressing cells are observed at the same location of Pax6-positive cells. Besides, these cells also show immunoreactivity to radial glia markers (GFAP and BLBP) and proliferative activity (PCNA immunoreactivity). Studies with neural stem cell lines indicate that Sox2 is a marker of stem cells and maintain neural stem cells in a proliferative and undifferentiated state. These studies also show that a complete ablation of Sox2 expression produces a loss of proliferative capacity; in addition, low levels of Pax6 are necessary to sustain proliferation and bipolar morphology of neural stem cells, while inactivation of Pax6 reduce the proliferative capacity of stem cells (Sakurai and Osumi 2008; Sansom et al. 2009; Gómez-López et al. 2011).

Our results show that Pax6-expressing cells located in the ventricular zone of the ventrolateral pallium express *ScSox2,* show proliferative capacity (PCNA) and molecular hallmarks of radial glia (GFAP and BLBP expression), as the mammalian neural stem cells present in the pallial ventricular zone.

### Neurogenic markers related with the differentiation of glutamatergic cell lineage are expressed in the developing OB of catshark

In the developing neocortex of mammals, the transcription factor Pax6 is expressed sequentially together with other transcription factors (Tbr2 and Tbr1) in progenitor cells and postmitotic neurons of glutamatergic cell lineage (Englund et al. 2005; Hevner et al. 2006). Interestingly, the transcriptional program that operates in the differentiation of glutamatergic phenotype in the developing neocortex is also necessary for correct morphogenesis of the OB and generation of mitral cells (Bulfone et al. 1998; Imamura and Greer 2013; Kahoud et al. 2014; Roybon et al. 2015).

In rodents, mitral cells are the first cellular subtype of the OB to be born; these cells are generated around the embryonic day E12 from progenitor cells located in the ventricular zone of the OB (Hinds 1968). Progenitor cells of mitral cells express Pax6 (Winpenny et al. 2011; Imamura and Greer 2013), and in Pax6 mutant mice mitral cells are misallocated (for review see: Nomura et al. 2007). Tracking experiments in mammals with BrdU labeling show that mitral cells are generated from Pax6-positive radial glial cells and post-mitotic mitral cell precursors also express both Tbr1 and Tbr2 during embryogenesis (Bulfone et al. 1999; Faedo et al. 2002; Imamura and Greer 2013).

Expression of both transcription factors is essential for the generation of mitral cells and its expression occurs in the same developmental period in which Pax6 expression is down-regulated (Imamura and Greer 2013). In Tbr2 mutant mice, the amount of mitral cells is reduced and their organization and projections disturbed, which is similar to what happens in Tbr1 mutant mice (Bulfone et al. 1998, 1999; Imamura and Greer 2013; Kahoud et al. 2014). Moreover, the bHLH transcription factor NeuroD is expressed in the mature glomerular layer, and overexpression of NeuroD leads to the appearance of mature neurons, but knockdown of NeuroD inhibits neuronal differentiation (Boutin et al. 2010). In amphibians, expression of Pax6, Tbr1 and Tbr2 is detected in the developing OB of embryos or larvae (Brox et al. 2004; Moreno et al. 2003, 2008) and Tbr1 expression is also present in the OB of zebrafish 48 hpf (Mione et al. 2001).

In the present work, we have detected *ScTbr2* and *ScNeuroD* expression and Tbr1 immunoreactivity in the primordial OB of S31 of development. Besides, a stream of Pax6-expressing cells can be seen coursing from the VZ of the pallium to the OP of S31 (present results; Ferreiro-Galve et al. 2012). Interestingly, the expression of Pax6 decreased in *ScTbr2-* and *ScNeuroD*-positive territories. In S32 embryos, the expression of *ScTbr2*, *ScNeuroD* and Tbr1 becomes restricted to territories adjacent to protoglomeruli; TH immunoreactive granular and periglomerular cells are also observed in the OB in late S32 embryos (Carrera et al. 2012; Quintana-Urzainqui et al. 2015). We did not find TH immunoreactivity in early S32 embryos indicating that at this developmental period, TH does not colocalize with Tbr1, which is in agreement with previous results in the catshark (Carrera et al. 2012; Quintana-Urzainqui et al. 2015). The expression pattern of neurogenic markers that we found at S32 embryos (present results), seems to be adjacent to the TH-positive cell populations described previously in the catshark, which suggest us that the territories expressing *ScTbr2*, Tbr1 and *ScNeuroD* may correspond to the prospective glomerular layer, where mitral cells are intermingled.

In addition, we have shown that both *ScTbr2* and *ScNeuroD* colocalize with Tbr1, but, as in rodents, it seems that some cells express Tbr1 or Tbr2 only (Imamura and Greer 2013). In the mammalian developing OB, studies of expression of Tbr2 and Tbr1 show that mitral cells express these factors in an overlapping pattern (Imamura and Greer 2013), which is in agreement with our results. On the other hand, in mammals, NeuroD expression pattern also overlapped Tbr2 expression, but not Tbr1 expression, indicating the existence of subsets of mitral cells (Roybon et al. 2015). Besides, recent studies show that maturation of mitral cells depends on their position in the developing OB (Nguyen and Imamura 2019). Some studies in elasmobranchs based on Golgi staining have evidenced the existence of two kind of mitral cells in the OB of the sharks studied (*Sphyrna tiburo, Dasyatis sabina* and *Rhizoprionodon terranovae)*: mitral cells with dense and tight arborized dendrites and mitral cells with a loose dendrite arborization (Dryer and Graziadei 1993). As far as we know, the existence of more than one type of mitral cells has not been described in *S. canicula* and in the present work, we were not able to define different subsets of mitral cells with the same markers used in mammals. Whether our results are in line with different degrees of maturation deserves further investigations.

In the catshark, the expression pattern of the different neurogenic markers overlapped with that of *ScvGlut1* (present results). In later embryos of rodents, Vglut1 was detected in the mitral cell layer (Ohmomo et al. 2011) and a strong expression of this transporter was also observed in mitral cells of adult reptiles (Sarkar and Atoji 2018).

**Fig. 5:**
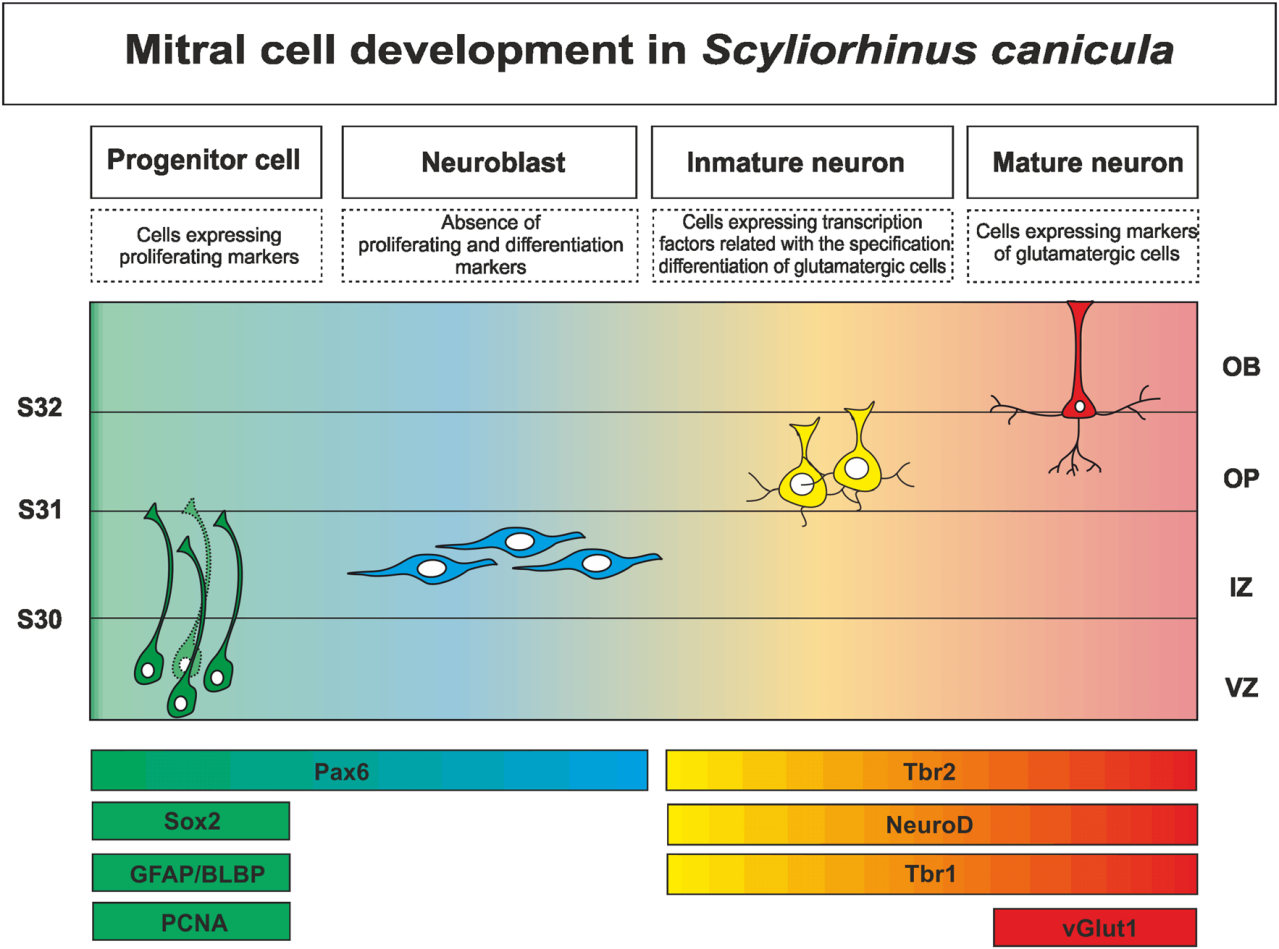
Scheme summarizing the expression pattern of glutamatergic neurogenic markers implicated in mitral cell development. Cell types and their molecular hallmarks are indicated on the top of the figure. Molecular markers used in the present study are indicated below, embryonic stages of development are represented as horizontal lines and indicated in the left, and anatomical regions are indicated in the right

All these findings together suggest us that Pax6-positive cells (with molecular hallmarks of radial glial cells; see above) that are present in the pallial ventrolateral ventricular zone generate new-born neuroblasts (Pax6 positive) forming a stream of Pax6-positive cells from the ventrolateral pallium to the OP. When these Pax6-expressing cells reach the OP, it seems that they experiment a downregulation of Pax6 expression and begin to upregulate transcription factors related with the specification/differentiation of glutamatergic cells (*ScTbr2, ScNeuroD*, Tbr1); later in development, when these molecular markers are still expressed, cells begin to express a marker of glutamatergic mitral cells (*ScvGlut1*) indicating that a maturity stage has been achieved (for a summary see Fig. 5).

In addition, mitral cells in the developing OB of catshark express the same transcription factors related with the differentiation of pallial glutamatergic cell lineage (Docampo-Seara et al. 2018a), suggesting that the transcriptional program which rules glutamatergic neurogenesis in different telencephalic areas has been conserved throughout the vertebrate evolution.

### The OB of mammals and sharks shares similarities in their developmental timing and molecular programs

Mitral cells are the first cell type to be born in the mammalian OB. These cells originate from progenitor cells located in the pallial/olfactory ventricular zone before the olfactory axons induce the emergence of the OP (E12) (Gong and Shipley 1995) and reach the OB following a radial migration process. Their neurogenic timing comprises from E10 to E13, with a neurogenic peak around E11 (Blanchart et al. 2006).

In the catshark, our results indicate that mitral cells are produced in the pallial VZ before the emergence of the OP. In the present work, we have performed BrdU pulse-chase experiments in catshark embryos before the emergence of the OB (pulse in 3 catshark embryos at S28, S29 and S30 and chase at S31, when the OP and markers of mitral cells are present). We found that most mitral cells are generated by progenitor cells that have divided at S28. However, some of them are also generated at S29. In S30pulse-S31chase BrdU-positive cells in the mitral cell territory are practically inexistent. This suggests that mitral cells are generated between S28 and S29. However, we cannot discard the possibility that some mitral cells could be generated at S30 (or later) and arrive to the OB later than at S31 of development.

When mitral cells are generated, in contrast to mammals, they seem to migrate tangentially up to S31; later, at S32, a diffuse band of glutamatergic cells is present in the primordium OB, which reminds their mature disposition in the adult OB.

On the other hand, in mice, interneurons are generated after the first waves of mitral cells arrive to the OB. Around E13.5, the subpallial lateral ganglionic eminence (LGE), begins to express the distal-less homeobox gene 2 (Dlx2) and, most part of OB interneurons is produced at this embryonic time (Wichterle et al. 2001; Vergaño-Vera et al. 2006; Kohwi et al. 2007). Interneuron production is persistent through the entire life of mammals, but during development, an important neurogenic peak can be detected between E15.5 and 17.5 (Batista-Brito et al. 2008; Lledo et al. 2008). During embryonic development, interneurons follow a tangential migration to the OB and, once they achieve the OB, they migrate radially and integrate in the OB circuitry. First interneurons from LGE arrived at the OB around E14.5 (Yoshihara et al. 2005).

**Fig. 6:**
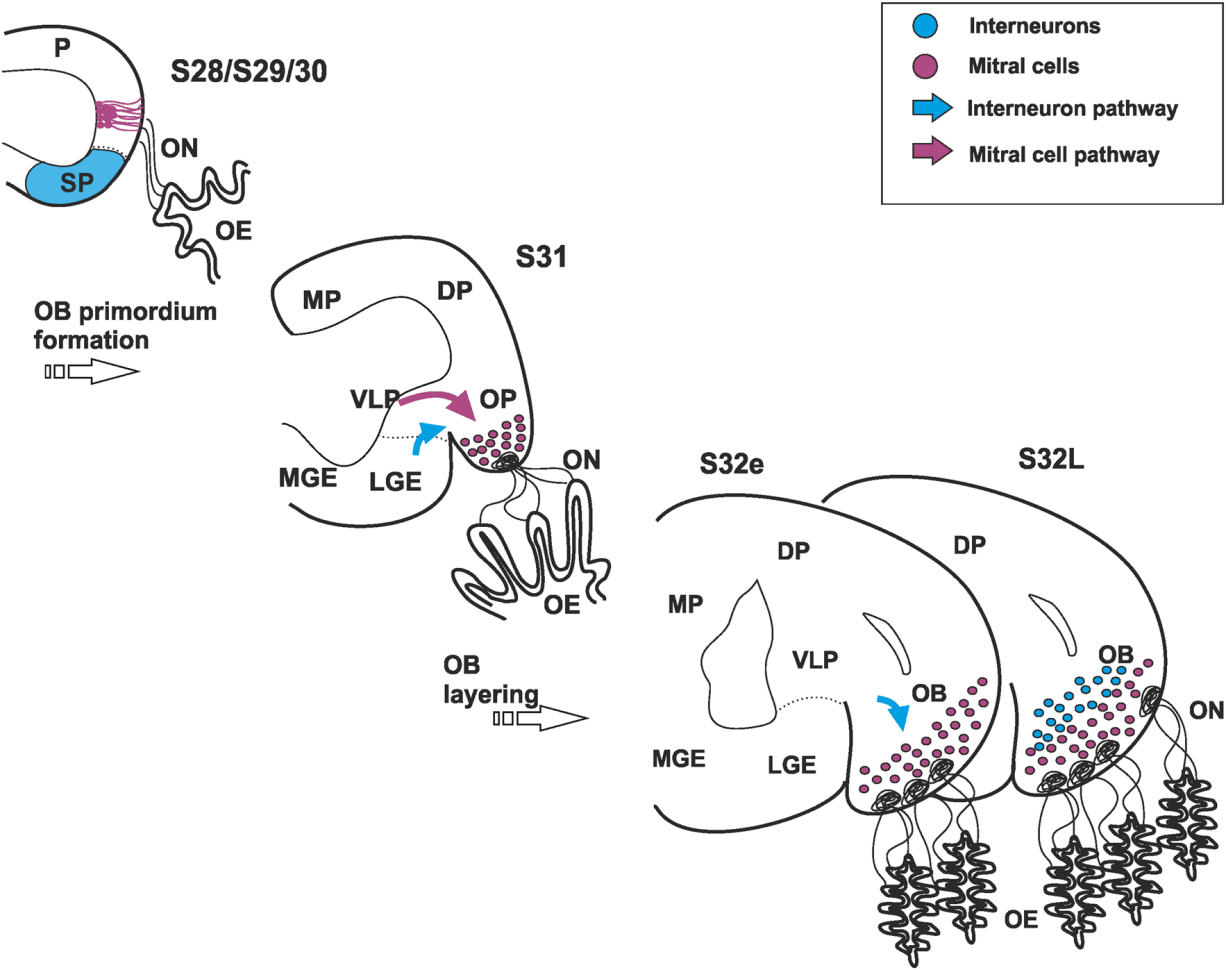
Scheme summarizing an integrated view of development of both mitral cells (present results) and interneurons in the OB of catshark (data from Quintana-Urzainqui et al. (2015) and Rodríguez-Moldes et al. (2017)

As in mammals, in the telencephalon of late embryos of catshark, a subventricular lateral stream of cells positive for *ScDlx2* directed towards the olfactory bulb have been observed at S31 (Quintana-Urzainqui et al. 2015). These cells originate in the subpallial lateral ganglionic eminence homologue in sharks (LGE-h; Quintana-Urzainqui et al. 2012) from S29 on (Quintana-Urzainqui et al. 2015). After this stream reaches the OB, numerous TH-ir cells are found in the OB from late S32 embryos onwards, which indicate that these cells are the source of the granular and periglomerular cells of the mature OB (Ferreiro-Galve et al. 2012; Carrera et al. 2012; Quintana-Urzainqui et al. 2015). For an integrated view of development in the catshark of both mitral cells and interneurons, see Fig. 6.

Equivalences between different embryonic stages of rodents and sharks have been made according to different developmental key events and three periods have been established (see Table 1 in Rodríguez-Moldes et al. 2017). Stages of development comprised between E10 and 14.5 of mice and S27–31 of catshark are comparable (second period; around 30 days in the catshark), while E14.5–20 of mice corresponds to S32–34 of catshark (third period; up to 100 days in the catshark). According to this, mitral cells are born and arrive to the OP of mice and sharks in the same developmental period. In addition, neurogenesis of dopaminergic neurons (granule and periglomerular cells) in sharks and mice seem to be comparable too. However, in the catshark, we cannot discard the possibility that generation of mitral cells and interneurons could be overlapped in development due to the long protracted embryogenesis of the catshark. An integrative view of both processes shows that the OB of mammals and sharks share similarities not only in their developing molecular programs, but also in the time of generation of projection neurons and interneurons.

### The presence of an accessory component in the OB of the catshark: an unsolved question

Most amphibians, reptiles and mammals possess a dual olfactory system with two separate neural pathways: the main olfactory system and the olfactory accessory system. The accessory olfactory system (or vomeronasal system; VNS) consists in a vomeronasal olfactory epithelium (VNO or Jacobson´s organ), an AOB, and the axons of the projecting neurons that reach different regions of the amygdala; from these region projections to hypothalamic regions are observed (for review see: González et al. 2010; Maximino et al. 2013).

Although in fish a unique olfactory epithelium is present and an AOB anatomically distinct of the MOB is not recognizable, an accessory olfactory system comparable to the VNS of tetrapods has been described in the lungfish using antibodies against transcription factors and neuronal markers characteristic of distinct portions of the VNS (González et al. 2010). In the catshark, ciliated sensory neurons, the typical cell morphology of the main OE, are lacking, and the main receptor neurons of the olfactory mucosa of *Scyliorhinus canicula* are the microvillous receptor neurons and crypt neurons, whose projections show a segregated distribution in the OB (Theisen et al. 1986; Ferrando et al. 2009). Genomic studies indicate that VNS-specific genes are present in teleost fish (for review see: Grus and Zhang 2009), cartilaginous fish (Sharma et al. 2019) and sea lamprey (Grus and Zhang 2009). In the catshark, V2R, a component of the vomeronasal signaling pathway, are the predominant chemosensory receptor family, and a small number of genes for chemosensory receptors of the main olfactory system are also present (Sharma et al. 2019).

In rodents, projection neurons of the MOB and anterior AOB originate from the same pallial ventricular zone. However, projection neurons of the posterior AOB arise from caudal areas located at the diencephalic–telencephalic boundary (Huilgol et al. 2013; Huilgol and Tole 2016). In the present work, we found a migratory stream from the pallial neuroepithelium toward the OB, but we do not have observed any evidence of a migratory stream from the diencephalic–telencephalic boundary towards the OB. Numerous studies in rodents indicate that Pax6 is required for OB mitral cell specification and differentiation in the MOB and anterior AOB (Huilgol and Tole 2016). In addition, Tbr1 is also involved in the specification of the projection neurons of the anterior AOB but not in the mitral cells of the posterior AOB (Huilgol and Tole 2016). However, in amphibians Tbr1 is also implicated in the migration of posterior AOB cells (Huilgol et al. 2013).

An important component of the vomeronasal system in rodents is the secondary bulbar projections. In rodents, mitral cells of the AOB project to the medial amygdala and from this region to the hypothalamus (Halpern and Martínez-Marcos 2003). In the lungfish, a region homologue to the medial amygdala of tetrapods expresses the transcription factor orthopedia (Otp) and Islet1 (ISL1), and different neuronal markers such as nitric oxide synthase (nNOS) and substance P (SP), which is in agreement with results obtained in amphibians (for review see: Maximino et al. 2013; González et al. 2010). In a previous study in the catshark, it was suggested that in S32 embryos, a lateral subpallial region with Pax6-, Dlx2- and GAD-expressing cells represent an amygdala-like structure, and this region in the adult brain shows numerous SP-immunoreactive fibers (Rodríguez-Moldes et al. 1993; Quintana-Urzainqui et al. 2012). In addition, after DiI application to the OB of juvenile of *Scyliorhinus canicula*, retrogradely labeled cells and fibers were observed in the dorsolateral part of the basal superficial area (a LGE-h derivative in the catshark, Quintana-Urzainqui et al. 2012) and in the lateral hypothalamus (Yáñez et al. 2011). However, further investigations are necessary to elucidate whether the lateral subpallial region in the telencephalon of catshark could be homologue to some amygdaloid territories of tetrapods.

Our results indicate that transcription factors (Pax6 and Tbr1) related with the specification of the MOB and anterior AOB of mammals are present in the catshark. Although we have observed regional differences in the combined expression of Tbr1 and Tbr2 in the OB of catshark, this observation does not allow us to reach any conclusion about the existence of an accessory component in the OB of the catshark. On the other hand, genomic and other cytological data (for review see: Ferrando and Gallus 2013: Sharma et al. 2019) indicate that the vomeronasal-type receptors component are the predominant in the olfactory epithelium, whereas main olfactory receptors are minority; moreover, ciliated olfactory neurons were not found in the olfactory epithelium of catshark, which suggest that the olfactory mucosa of catshark may be similar to the vomeronasal epithelia of tetrapods. Further, studies of the expression/pattern of transcription factors and neuronal markers related with the specification/differentiation of different components of the vomeronasal system in the central nervous system are key to elucidate the existence of the brain areas/connections implicated in the processing of vomeronasal-type information in the catshark.

## Conclusions

In the present work, we have carried out a detailed analysis of the transcription factors (Pax6, Tbr2, NeuroD and Tbr1) involved in the glutamatergic neurogenesis during OB development in the catshark. Proliferative Pax6-positive cells with stem cell and radial glial features are present before the emergence of the OB in the pallial ventricular zone of catshark. Cell tracking analysis using BrdU confirms that progenitors with these characteristics give rise to cells that migrate to the OP and locate in the presumptive territory where glutamatergic/mitral cells should be located. We found that mitral cells in the catshark seem to express the same battery of transcription factors found in mammals, which seem to indicate that the transcription program of glutamatergic cells specification/differentiation has been conserved throughout vertebrates evolution. However, the same factors that in mammals clearly differentiate subsets of mitral cells (Tbr2-Tbr1, Tbr2-NeuroD) do not differentiate them in the catshark.

In addition, comparisons between our data and previous studies have evidenced that the origin and expression pattern of transcription factors related with the specification/differentiation of glutamatergic are common to mammals, evidencing a high conserved pattern of neurogenesis.
